# Dominance rank, facial morphology and testes size in male white-faced capuchins: evidence for pre- and post-mating competition

**DOI:** 10.1098/rspb.2025.0645

**Published:** 2025-08-20

**Authors:** Nicholas Chapoy, Marcos Alvarez-Garita, Evelyn Howard, Blanca Cejalvo Insausti, Nelle K. Kulick, Mercedes Marinaro, Hubert A. Mendez, Suheidy Romero Morales, Megan Petersdorf, Alice C. Poirier, Robinson Sandoval, Giulia Severino, Wendy Téllez Arias, Anja Widdig, Amanda Melin, Katharine Jack

**Affiliations:** ^1^ Department of Anthropology, Tulane University, New Orleans, LA, USA; ^2^ Santa Rosa Primate Conservation Fund, Área de Conservación Guanacaste, Guanacaste, Costa Rica; ^3^ Insituto Internacional en Conservacion y Manejo de Vida Silvestre, Universidad Nacional de Costa Rica, Heredia, Costa Rica; ^4^ Department of Anthropology and Archaeology, University of Calgary, Calgary, Alberta, Canada; ^5^ Fundació UdG: Innovació i Formació, University of Girona, Girona, Spain; ^6^ Institute of Biology, Universitat Leipzig Fakultat fur Biowissenschaften Pharmazie und Psychologie, Leipzig, Germany; ^7^ Department of Primatology, Max Planck Institut for Evolutionary Anthropology, Leipzig, Germany

**Keywords:** photogrammetry, intrasexual dimorphism, testicular morphology, facial width-to-height ratio, sperm competition

## Abstract

Male reproductive success is determined by the interplay of female mate choice and male–male competition, often linked to dominance rank in social animals. Across taxa, elaborate ornaments, such as bright coloration or large antlers, often function as badges of status, signalling male competitive ability to rivals. In species where females mate with multiple males, post-mating sperm competition also plays an important role in male reproductive success and is associated with larger relative testes size. We investigate the relationship between morphological features and dominance rank in wild male white-faced capuchins. Using parallel-laser photogrammetry, we measured aspects of facial morphology, including facial width-to-height ratio, and testes size. We found that alpha males had significantly larger facial width-to-height ratios, wider faces and wider scrota than subordinate males. These results suggest that facial traits potentially function as badges of status in male white-faced capuchins and may play a role in pre-mating competition and/or mate choice, while differences in scrotal size reflect adaptations for post-mating competition. This study highlights the under-recognized role of facial trait evolution in sexual selection among relatively gracile yet highly visually oriented mammals and the potential variability of sexual traits in species characterized by strong reproductive skew among males.

## 1. Introduction

Dominance hierarchies are a fundamental component of social organization in most group-living mammals, often characterized by an asymmetric distribution of access to resources among a small number of individuals ([1–8], reviewed in [9]). In species with intense male–male competition, male dominance rank is primarily established through agonistic confrontations [10–12], which can come with significant costs to participants, including expulsion from the social group, severe injuries and, in extreme cases, death [6,13–18]. Given these potential costs, the benefits of attaining a high dominance rank must be considerable. In many species, high-ranking males often gain preferential access to food [8,19,20] and mating opportunities [7,21–26], resulting in these males siring a disproportionate percentage of offspring (e.g. [21,27–29] reviewed in [30,31]).

To mitigate the risks associated with attaining and maintaining high dominance rank, animals across diverse taxa have evolved signals to advertise male quality and/or individual competitive ability, which may include visual, auditory, chemical and/or vibrational traits (primates: [32–37]; other mammals: [38–43]; birds: [44–48]; insects: [49–51]; amphibians: [52]; reptiles: [53–55]; reviewed in [9,56,57]). Visual signals that involve the development of exaggerated secondary sexual characteristics, referred to as ‘badges of status’ [58,59], can enable males to assess opponents and determine whether to engage in a conflict or to withdraw and avoid costly physical confrontations [6,45,60,61]. Examples of badges of status can include conspicuous visual signals such as the bright coloration of faces and genitalia of dominant male mandrills (*Mandrillus sphinx:* [14]) and the elaborate trains of peacocks (*Pavo cristatus*: [23]).

In species where male dominance rank is primarily determined by male–male contest competition, dominant males often experience elevated testosterone levels (e.g. [62–64]). Testosterone plays a key role in the development of secondary sexual traits and, thus, in the elaboration of badges of status ([65,66], primates reviewed in [67]). For example, elevated testosterone levels are linked to growth of beards in humans [68], the cheek pads, throat pouch and long hair of orangutans (*Pongo* spp.) [64] and the size of chest patch in house sparrows (*Passer domesticus*) [69,70]. Testosterone also affects post-mating competition [30,71]. In species with high levels of sperm competition (i.e. where females mate with multiple partners), males with larger testes tend to achieve greater reproductive success [72,73]. This is because larger testes enable the production of greater quantities of higher-quality sperm ([47,74–79] reviewed in [80]). In some species, the colour and/or size of male genitalia may play a dual role, contributing to both pre- and post-mating competition [30,78]. For example, in mandrills, achieving high social status is associated with increased testes size and enhanced genital redness [81]. These traits may serve as signals of dominance, potentially deterring rival males, much like the display of large canines, or can make males more attractive to females. Simultaneously, increased testes size can confer advantages in sperm competition, collectively enhancing their reproductive success.

Studies of badges of status have largely focused on species with highly conspicuous sexually selected traits, such as mane length and colour in lions (*Panthera leo* [42]) or the facial flanges of dominant male orangutans [82,83]. However, this emphasis may reflect anthropocentric biases, and subtler forms of badges of status may be more widespread than currently recognized. Expanding research to include less obvious forms will contribute to broadening the comparative framework for studies of sexual selection. Given the high reliance on vision in primates, investigating badges of status in a relatively gracile species with more understated traits offers a promising area for advancing our understanding of these signals.

Here, we examine male facial and scrotal morphology in a species of wild primates, white-faced capuchins (*Cebus imitator*), to assess if and how they vary with male dominance rank. These primates belong to the gracile radiation of capuchins [84] and live in multi-male, multi-female social groups characterized by female philopatry and male dispersal [85]. White-faced capuchins are a sexually dimorphic species, with adult males being approximately 45% heavier and possessing canines around 60% longer than adult females [86–89]. Male–male competition over group membership and the attainment of alpha rank status is intense in this species [90] and can result in severe injury or death for competing males [13,91,92]. However, once the male dominance hierarchy stabilizes, agonism among males within social groups is rare [93,94]. Although subordinate males may occasionally reproduce, the reproductive benefits of attaining alpha male rank status are substantial. Alpha males sire most of the offspring born during their tenure (*ca* 80–100%), which can last from a few weeks to as long as 18 years [95–98]. This reproductive skew is probably influenced by traits enhanced by elevated androgen levels in alpha males compared with subordinate males, which can improve mating success and thus contribute to the observed reproductive skew [99]. Although limited to a single case study in white-faced capuchins, it appears that male testosterone rises after attaining alpha status [100], which has also been reported for the closely related *Sapajus libidinosus* [101].

Early research on white-faced capuchins described alpha males as the largest individuals within a group, suggesting a direct correlation between overall body size and dominance rank status [97,102]. Subsequent studies noted that alpha males also appear to have more pronounced secondary sexual characteristics, particularly in their facial morphology. Specifically, alpha males were noted as having broader brow ridges and more developed muzzles and jowls than subordinate adult males [99,103,104], raising the potential for the presence of badges of status in this species. Despite anecdotal evidence linking physical size with dominance rank status in this species, there has been no formal quantification of this relationship. This gap limits our understanding of the evolutionary pressures that drive variation in morphological traits in capuchins and their potential roles in pre- and post-mating competition. In this study, we use parallel-laser photogrammetry to examine the facial morphology of wild male white-faced capuchins and assess its potential as a badge of status. We further measure scrotal size to assess the potential of this trait to be responsive to dominance rank status and reproductive effort. Finally, we include body length as a covariate in our analyses to control for the possibility that larger faces and scrotal sizes are merely by-products of overall larger body size.

## 2. Methods

### (a) Data collection

#### (i) Study site and subjects

We collected data on all males (6+ years old) residing in five habituated groups of white-faced capuchins (*C. imitator*) between January 2021 and May 2024 in the Santa Rosa Sector of the Área de Conservación Guanacaste, Costa Rica (SSR). This population of capuchins has been studied almost continuously since 1983 [105,106]. All males were individually identified by physical characteristics (e.g. cap/peak shape, scars, size, missing digits and other idiosyncrasies). Male ages were known, based on observed date of birth (*n* = 14 individuals), or estimated at first sighting based on comparisons with males of know ages (*n* = 8 individuals). In this species, males typically disperse from their natal group around 4.5 years of age (range: 1.7−8 years; [107]). Based on phenotype at the time of immigration and long-term comparisons with individuals of known-age individuals, we are confident in our ability to estimate the ages of immigrant males to within 1−2 years. During the study period, groups were followed at least twice per month, during which intensive behavioural and demographic data were collected. While white-faced capuchins form multi-male groups with a distinct alpha male and socially integrated subordinates, there is no clear linear dominance hierarchy among sub-alpha ranking males [103]. Therefore, males were categorized as either alpha (*n* = 10) or subordinate (*n* = 12), with alpha males readily identified based on behavioural traits and submissive behaviours from group mates [92,99,103]. Over the course of the study, the population experienced five alpha male replacements (AMR; i.e. changes in dominance rank). To minimize the impact of potential morphological changes related to these AMRs, we selected photographs exclusively from individuals in stable groups that had not experienced an AMR event for at least six months. Each male in this study represents a single dominance rank (either alpha or subordinate).

#### (ii) Photograph collection

We used parallel-laser photogrammetry [108,109] to non-invasively measure male facial features, scrotal width and body length, as detailed below. We used a Nikon D3500 DSLR camera (24.2 MP resolution), with a Nikon AF-P DX Nikkor 70−300 mm f/4.5−6.3G ED IF lens or a Tamron AF 70−200 mm 2.8 IF lens, and a parallel-laser box mounted to the top of the camera using the multi-function shoe. We used two different parallel-laser apparatuses during this study. The design of the first apparatus, which was used from January 2021 to June 2023, was a custom-made laser box (Picotronic, Koblenz, Germany) housing class II lasers powered by an external battery pack [109]. Due to issues with poor durability under our field conditions, the cost and expense of repair, we transitioned to a custom-built parallel-laser apparatus constructed using commercially available parts (adapted from [110]). This apparatus was made up of class IIIA lasers mounted to the top of the camera and proved to be much more robust under our field conditions. Prior to each data collection day, the lasers for the first apparatus were calibrated to an inter-laser distance of 30.00 mm (error: ±0.2 mm), and the second was calibrated to 45.00 mm (error: ±0.2 mm) using the same protocol and digital calliper to measure the distance for both. For calibration, the lasers were first directed at a template taped to the wall and measured at 5 m. Once completed, this procedure was repeated at distances of 1–10 m to ensure that the lasers remained parallel. Although we were not able to apply both laser apparatuses to the same subjects for direct comparisons, each apparatus was rigorously calibrated to minimize potential discrepancies and to ensure consistent data quality across the study. To further validate the consistency of measurements between the two laser apparatuses, ‘apparatus type’ was included as a fixed effect in all our models. However, its inclusion did not significantly alter the results, and it was subsequently removed to simplify the analyses without compromising the integrity of the findings.

**Figure 1.**
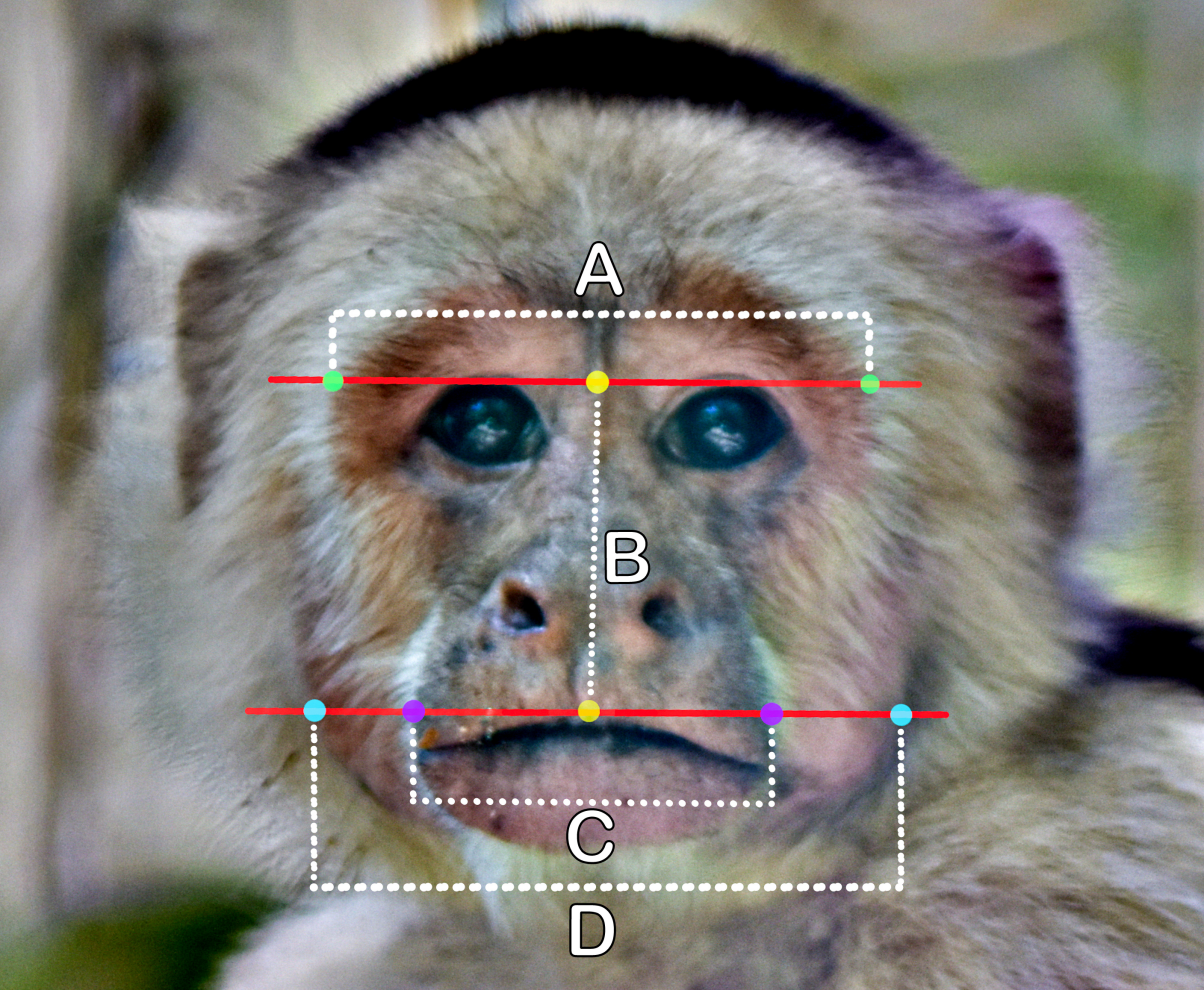
(A) Brow width—the lateral–medial width of the supraorbital torus, (B) facial height—the height of the face from between the eyes to the middle of the upper lip, (C) muzzle width and (D) facial width—the distance between the most lateral points of each side of the mandible. Facial width-to-height ratio (fWHR) was calculated as D : B.

**Figure 2.**
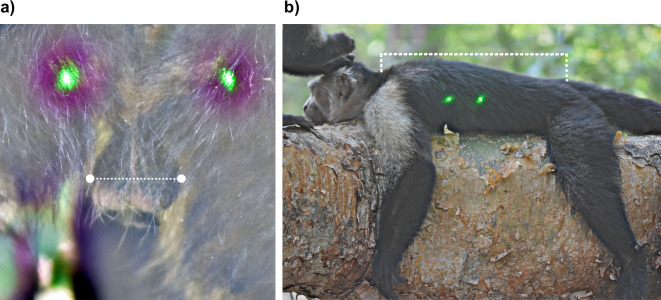
Scrotum width—the width across the widest part of the scrotum (a) and, (b) Body length—from the base of the neck to the base of the tail (i.e. where the tail meets the back).

For each male, we collected photographs (three to five each of the face, body length and scrotum) on two occasions during each three-month interval, across both the dry and wet seasons. The photographs were captured between 07.30 and 18.00. We used the following criteria when taking photographs in the field: (i) both lasers were visible, either projected onto the individual’s body (i.e. avoiding the face and eyes) or on an equidistant substrate; (ii) the physical feature of interest (i.e. face, body and scrotum) was completely visible and appeared as perpendicular to the axis of the camera to avoid parallax error [111]; (iii) facial features were only photographed when individuals were in neutral positions (i.e. eyes open, mouth relaxed, eyebrows neither furrowed nor raised; figure 1); (iv) photographs of the scrotum were taken from behind the individual, typically in a walking stance (figure 2); and (v) photographs of body length were taken when individuals were walking, standing or laying quadrupedally, with a straight back, and arms and legs perpendicularly under the body (figure 2b).

#### (iii) Photograph measurements

Photographs were analysed in the Jack Lab at Tulane University using a scoring system to assess their quality. Photographs were randomly assigned to observers (*n* = 2–5), who were blind to the social status of the monkey depicted in the photo. Observers assigned scores based on the standards defined above to determine measurability. A score of 1 indicated the photograph was of high quality with all criteria met, a score of 2 was given to a photograph that, while not perfect, was of sufficient quality for further analysis, and a score of 3 indicated the photograph did not meet criteria (i.e. lighting, exposure, blurriness and overall quality). A photograph needed to score 1 or 2 to be included in subsequent analyses. After scoring and filtering out those of poor quality (score of 3), a total of 513 photographs were measured and included in the analyses, including 213 photographs from 20 males (10 alphas and 10 subordinates) for facial features, 178 photographs of 19 males (8 alphas and 11 subordinates) for scrotum width and 122 photographs of 21 males (10 alphas and 11 subordinates) for body length. Selected photographs were uploaded in jpeg format to the GIMP GNU image manipulation program (v. 2.10.34), a free and open-source image editing program (https://www.gimp.org). We applied GIMP’s standard high contrast filter to each photograph, which enhanced the visibility of the morphological features of interest due to the black and white pelage of the study species. Following Richardson *et al*., we used the calibrated inter-laser distance (30.00 or 45.00 mm depending on the laser used for the photograph) divided by the inter-laser distance from the image in pixels (calculated using the ‘measure tool’ in GIMP), to create a millimetre per pixel scale [112].

We measured the following four facial features (figure 1): (i) brow width—i.e. the lateral–medial width of the supraorbital torus; (ii) facial height—i.e. the height of the face from between the eyes to the middle of the upper lip; (iii) muzzle width—i.e. the lateral width of the upper snout, measured across the maxilla just above the upper lip; and (iv) facial width—i.e. the width from the most lateral points of each side of the mandible, which is the widest visible portion of a capuchin male face. From these measurements, we calculated the facial width-to-height ratio (fWHR) by dividing facial width by facial height (figure 1, D : B). We also measured the width of the scrotum across its widest point (figure 2a). We initially attempted to measure the height of individual testes as well (e.g. [113]), but these measurements proved inconsistent due to the lack of contrast caused by the dark skin and dense hair in this area. Finally, we measured body length, from the base of the neck to the base of the tail (i.e. where the tail meets the back; figure 2b). Each laboratory assistant independently placed landmarks directly onto the photograph using the ‘paintbrush’ tool in GIMP. After placing the landmarks for each of the relevant features described above, each assistant measured inter-landmark distance in units of pixels using the ‘measure tool’. The inter-landmark distance (in pixels) was then multiplied by the previously calculated millimetre per pixel scale to produce a landmark feature estimate in units of millimetres (electronic supplementary material, figure S1; modified from [112]). To establish a final measurement for each photograph, the mean was calculated across all observers. Any obvious outliers were re-evaluated, re-measured by an additional assistant and/or removed from the dataset. To assess inter-observer reliability, we calculated the intra-class correlation coefficient (ICC) using the icc() function in the ‘irr’ R package [114] for each measured feature from a randomly selected subset of 50 photographs. All ICC values exceeded 0.80 (electronic supplementary material, table S1). These results indicate high inter-observer reliability, suggesting that the morphological landmarks are consistently identifiable and that observers are able to locate them reliably.

### (b) Statistical analyses

We first performed a principal component analysis (PCA) using the principal() function from the ‘psych’ package [115] in R (v. 4.4.2 [116]) and RStudio (v. 2024.9.1.394 [117]) to assess the relationships among facial measurements (facial width, brow width, muzzle width and facial height) and to check for multi-collinearity. Prior to the PCA, we checked for sampling adequacy (Kaiser–Meyer–Olkin test: measure of sampling adequacy = 0.66) and sphericity (Bartlett’s test: *χ*^2^(6) = 49.75, *p* < 0.001) to ensure the appropriateness of our data for factor analysis. The data were standardized prior to the PCA using the scale() function, and the PCA analysis was conducted with a varimax rotation to enhance the interpretability of the principal components. The PCA identified four principal components, each accounting for approximately 25% of the total variance (electronic supplementary material, table S2). Each facial measurement loaded strongly onto a separate principal component, indicating that each measurement captured distinct aspects of morphological variation. Based on these findings, we analysed each facial measurement independently.

To account for individual differences in overall body size, we included body length (in mm) as a covariate in our analyses. However, because photographs were taken intermittently and often not on the same day as photographs of other body parts, some body length data were missing. To address these missing values and maintain the temporal integrity of the repeated measures within individuals, we used an imputation method that replaced missing body length values with the closest available measurement in time. Although the range of days between body length measurements was considerable (1–436 days), the monkeys in our study were fully mature, and their body length is unlikely to change significantly over time.

Six separate linear mixed models (LMMs) were fit individually for scrotum width, facial width, muzzle width, brow width, facial height and fWHR using the ‘lmer’ function from the ‘lme4’ package [118]. Dominance rank (alpha/subordinate), body length (in mm) and age (in years) were included as fixed effects, while individual identity was included as a random effect to account for repeated measures from the same individuals. Each response variable followed a normal distribution (Shapiro–Wilks tests, *p* > 0.05). To further evaluate the significance of the fixed effects, we used the drop1() function to assess the contribution of each predictor to the model by comparing nested models. Conditional *R*^2^ values were estimated using the r.squaredGLMM() function from the ‘MuMIn’ package [119]. Assumptions for each model were checked using the ‘DHARMa’ package [120]. Homogeneity of variance was assessed by examining residual versus fitted plots, ensuring consistent variance across predictor levels. Residuals were tested for normality using visual inspection of *Q*–*Q* plots and confirmed by the Kolmogorov–Smirnov test, which indicated no deviations from normality. Additionally, no models exhibited signs of overdispersion (range across all models: 0.90–0.97), confirming the adequacy of the model fit.

**Figure 3.**
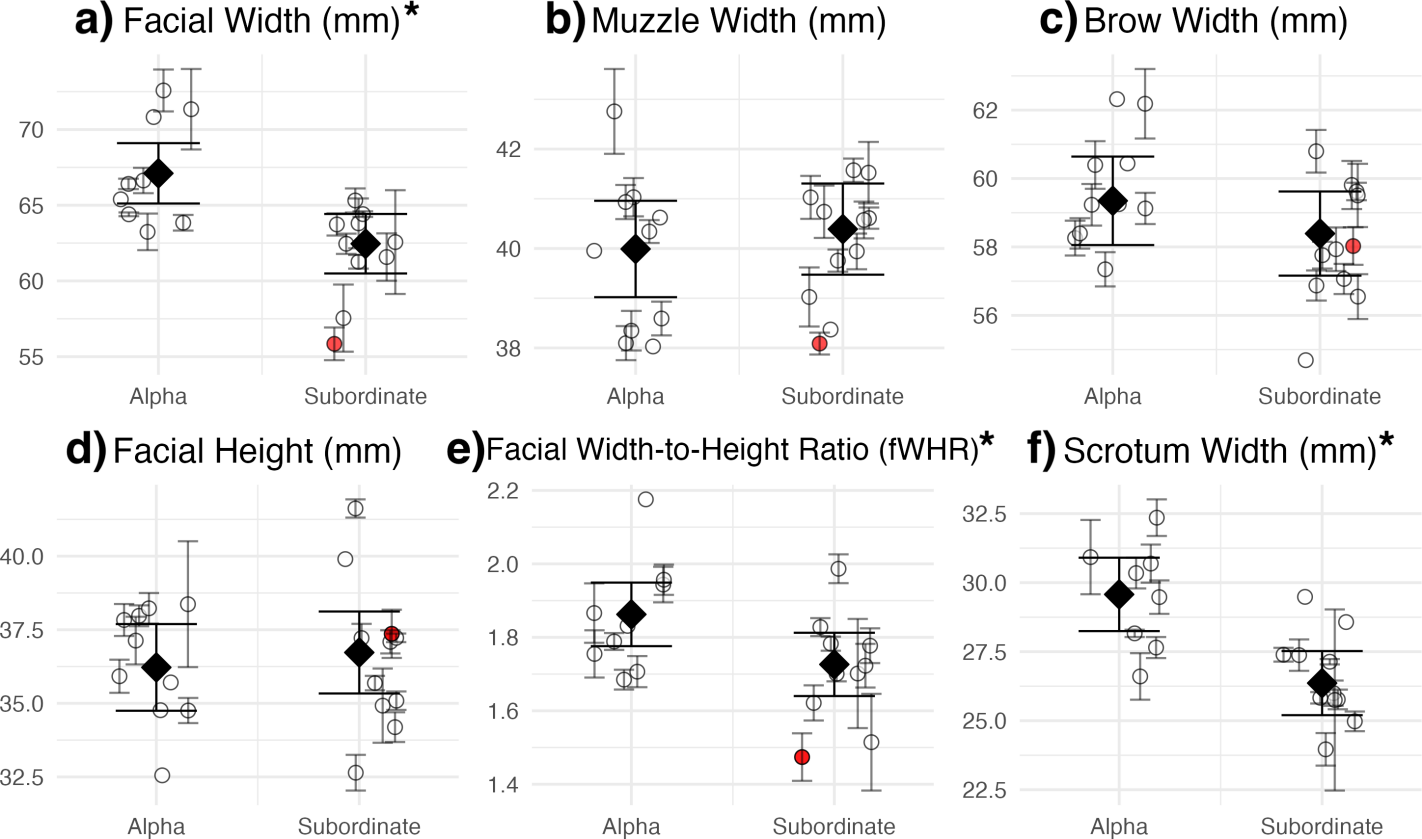
Relationship between dominance rank and (a) facial width, (b) muzzle width, (c) brow width, (d) facial height, (e) FWHR and (f) scrotum width. Each point represents a single individual’s mean value, with error bars indicating the standard error across multiple measurements. Model-predicted group means with 95% confidence intervals are overlaid. The outlier is shown in red and was excluded from model fitting. Asterisks (*) indicate significant associations with dominance rank.

**Figure 4.**
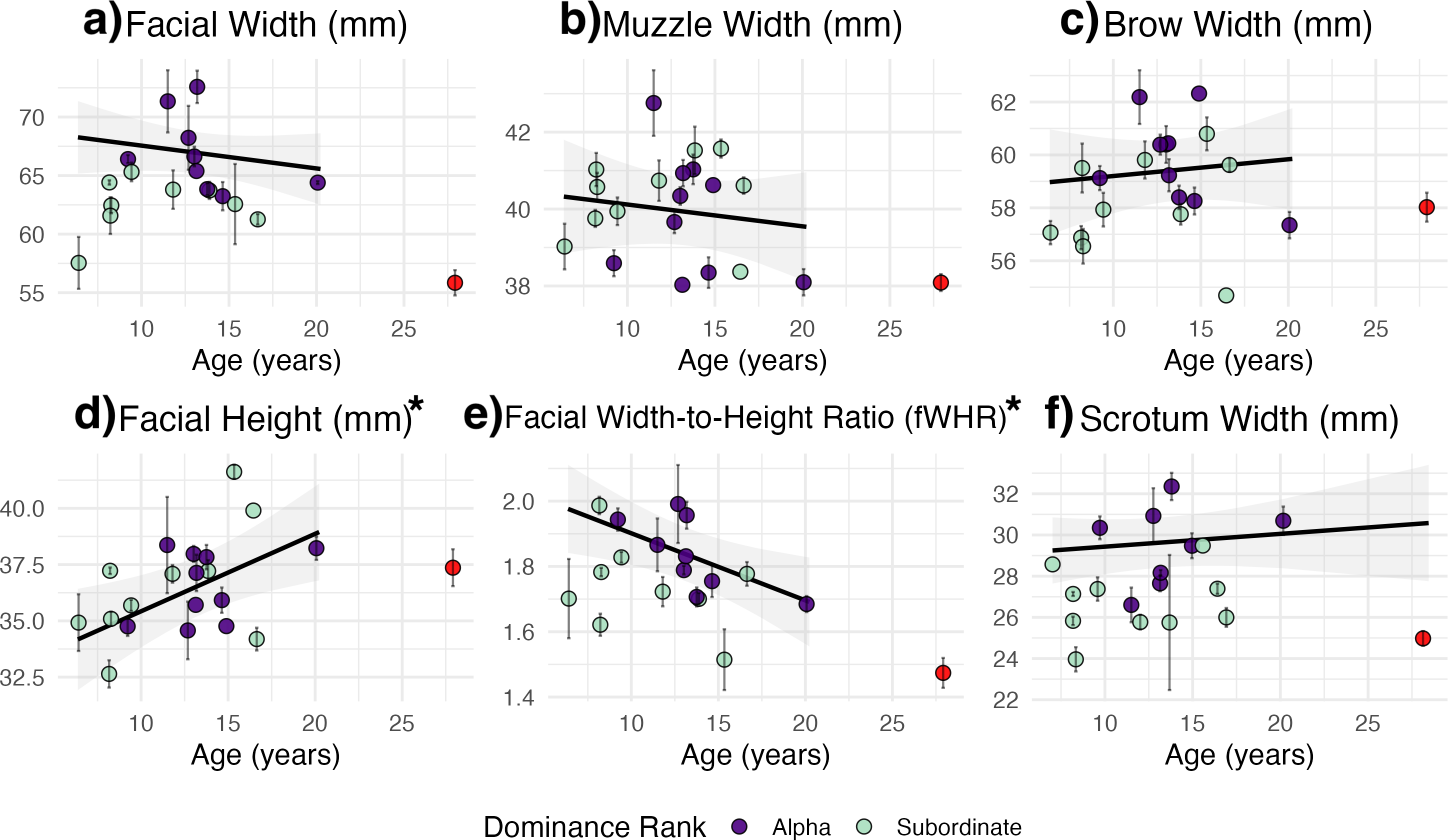
Relationship between age and (a) facial width, (b) muzzle width, (c) brow width, (d) facial height, (e) FWHR and (f) scrotum width. Each point represents a single individual’s mean value, with error bars indicating the standard error across multiple measurements. Model-predicted values across age with shaded 95% confidence intervals are overlaid. The outlier is shown in red and was excluded from model fitting. Asterisks (*) indicate significant associations with age.

During data analysis and visualization, we discovered that the facial measurements of an exceptionally old (28 years) subordinate male emerged as extreme outliers when considering how age influences morphology and may be driving our results (the next oldest male in our sample was 20 years at the end of the study; mean age = 13.1). Inclusion of this individual substantially influenced the results, creating a significant negative correlation between age and facial width that disappeared when his data were removed. Since this statistical relationship was determined by just this one male, we opted to exclude him from our main analyses to prevent basing conclusions on an outlier case. For complete transparency, we have included the results of analyses with this outlier male in the electronic supplementary material, table S3. This individual is represented in red in figure 3 and figure 4, to distinguish him from the rest of the sample but allow visual assessment of his influence. This male remained in the scrotal measurement results, as his scrotal width measurements fell within the normal range of variation for the population, and his exclusion did not change the results.

## 3. Results

Our analyses revealed distinct patterns in morphological features related to dominance rank and age, independent of body length. Specifically, facial width and scrotum width were significantly larger in alpha males but did not vary with age (table 1; Figure 3 and figure 4). The fWHR was also significantly larger in alpha males and inversely related to age, with younger males exhibiting relatively shorter and wider faces (table 1; Figure 3 and figure 4). Facial height did not differ by dominance rank but was significantly larger in older males (table 1; Figure 3 and figure 4). Muzzle width and brow width showed no significant relationships with either age or dominance rank (table 1; Figure 3 and figure 4). These relationships in facial and scrotal dimensions were independent of body length, which was not a significant predictor of any of the measured morphological traits (table 1).

**Table 1.** Results of LMMs testing whether (a) facial width, (b) muzzle width, (c) brow width, (d) facial height, (e) facial width-to-height ratio, or (f) scrotum width are predicted by dominance rank, overall body size and/or age. Significant *p*-values are bolded.

explanatory variable	effect size	s.e.	*c*^2^ (d.f.)	*p*
**(a) facial width model. Model conditional** ***R***^**2**^ **= 0.41; alphas** ***n*** **= 9, subordinates** ***n*** **= 9**				
intercept	79.22	11.78	—	—
dominance rank (subordinate versus alpha)	−4.65	1.33	11.12 [1]	**0.0009**
body length	−0.04	0.05	1.25 [1]	0.26
age	−0.20	0.18	1.44 [1]	0.23
**(b) muzzle width model. Model conditional *R*^2^ = 0.44; alphas *n* = 10, subordinates *n* = 10**				
intercept	38.03	4.63	—	—
dominance rank (subordinate versus alpha)	0.40	0.64	0.55 [1]	0.46
body length	0.01	0.02	0.18 [1]	0.67
age	−0.06	0.08	0.35 [1]	0.55
**(c) brow width model. Model conditional *R*^2^ = 0.44; alphas *n* = 10, subordinates *n* = 10**				
intercept	49.35	6.48	—	—
dominance rank (subordinate versus alpha)	−0.96	0.86	1.52 [1]	0.22
body length	0.04	0.02	1.17 [1]	0.28
age	0.06	0.11	0.29 [1]	0.59
**(d) facial height model. Model conditional *R*^2^ = 0.57; alphas *n* = 10, subordinates *n* = 10**				
intercept	27.57	6.56	—	—
dominance rank (subordinate versus alpha)	0.51	0.98	0.24 [1]	0.62
body length	0.02	0.02	0.45 [1]	0.50
age	0.34	0.13	7.54 [1]	**0.006**
**(e) facial width-to-height ratio model. Model conditional *R*^2^ = 0.45; alphas *n* = 9, subordinates *n* = 9**				
intercept	2.49	0.47	—	—
dominance rank (subordinate versus alpha)	−0.14	0.06	6.37 [1]	**0.01**
body length	−0.001	0.002	0.89 [1]	0.35
age	−0.02	0.008	1.30 [1]	**0.007**
**(f) scrotum width model. Model conditional *R*^2^ = 0.66; alphas *n* = 8, subordinates *n* = 11**				
intercept	33.88	5.14	—	—
dominance rank (subordinate versus alpha)	−3.22	0.82	12.23 [1]	**0.0005**
body length	−0.02	0.02	0.94 [1]	0.33
age	0.06	0.08	0.48 [1]	0.49

## 4. Discussion

In this study, we used parallel-laser photogrammetry to non-invasively measure the facial and scrotal morphology of male white-faced capuchins to evaluate whether and how these traits vary with male dominance rank. We found that dominance rank (subordinate or alpha) significantly predicts facial width and fWHR, with alpha males exhibiting significantly wider faces and larger fWHRs compared with subordinate males. Interestingly, body length did not significantly predict variations in facial features or scrotum width, suggesting that larger overall body size does not necessarily correlate with larger dimensions in the features analysed. This challenges the assumption that overall size correlates with dominance rank and suggests that facial traits may function as badges of status [121]. However, body mass and muscle mass, factors that were not measured in this study, may still influence dominance and reproductive success (e.g. [122]) and should be considered in future research. While badges of status can signal male quality and influence mate attraction, our findings that these traits vary according to male dominance rank status, which is competitively determined in this species, suggest that they are primarily shaped by male–male competition. However, it is possible that both males and females respond to these signals for different evolutionary reasons, indicating a potential role for female mate choice as well (e.g. [123,124]). Such visual signals may help to minimize the frequency of costly physical confrontations, potentially explaining the low levels of male–male intra-group aggression in this species [99]. These results are consistent with findings across a number of other primates, where dominant individuals consistently exhibit larger facial morphologies compared with subordinates (*Homo sapiens*: [125,126]; *Macaca* spp.: [127]; *Pan paniscus*: [128]; *Pan troglodytes* (females only): [129]; *Sapajus* spp*.*: [130,131]; reviewed in [132]). However, further studies are essential to empirically validate whether facial width influences male capuchin behaviour, particularly to determine if males with wider faces and larger fWHR elicit higher rates of submission or avoidance from conspecifics.

At the proximate level, the distinctive submandibular morphology of alpha males probably results from increased androgen receptor activity, which may drive bone growth and/or muscular hypertrophy in response to elevated levels of circulating androgens [133–136]. Similar androgen-related changes in male facial morphology have been reported in orangutans [61,64]. Another possible explanation is the activation of mandibular scent glands in this region, as seen in numerous primate species [137]. For example, neck/mandibular rubbing is more common among males than females in both red-ruffed lemurs (*Varecia variegata rubra*) and brown howler monkeys *(Alouatta guariba*) and may be associated with intrasexual signalling of dominance rank status and/or androgen levels [138–140]. However, it remains unclear whether capuchins possess such glands [137,141], highlighting an area for future investigation.

We also found that age significantly predicts both facial height and fWHR, with younger males having larger fWHRs and older males having longer faces. These results suggest that facial morphology may continue to change across an individual’s lifetime, potentially reflecting shifting social roles, hormonal fluctuations and age-related degeneration (e.g. bone resorption, tooth loss and craniofacial remodelling), as observed by the aged male in our study. Such changes have been observed in other non-human primates (e.g. chimpanzees: [142]; Japanese macaques (*Macaca fuscata*): [136]). Given the relatively young age of the individuals in our study (mean age: *ca* 13 years), longitudinal data on a wider age range of individuals are needed to fully understand the impact of ageing on facial morphology in white-faced capuchins.

We examined scrotal width according to male dominance rank to assess the potential for dominance-related effects on male reproductive effort. We found that alpha males exhibit significantly wider scrotums than subordinate males. Within capuchin groups, most males experience some level of mating success, and overt male–male competition for mating opportunities within groups is rare [143]. Indeed, it is not uncommon for females to actively solicit and mate with subordinate males in full view of the group’s alpha male. While some males and females do occasionally sneak off from the group to copulate, sexual interactions in this species are usually very conspicuous, involving a coordinated duet between males and females that includes specific facial expressions (‘duck faces’), behaviours and vocalizations [144,145]. Despite the lack of overt intra-group male mating competition, alpha males sire the majority (*ca* 80–100%) of group offspring [95,96,146]. Previous studies suggested that this high reproductive skew is largely driven by female mate choice based on observations that females selectively mate with alpha males when they are cycling and with subordinate males when they are pregnant and/or lactating [147]. This mating pattern may function as a female strategy to reduce the risk of infanticide associated with AMR in this species (i.e. paternity confusion [90]). However, our finding that alpha males exhibit larger testicular volume (measured as scrotal width), probably driven by higher testosterone levels (e.g. [103]), suggests that post-mating sperm competition also plays a role in alpha male reproductive success and the resultant reproductive skew that characterizes this species. Larger testicular volume may provide alpha male capuchins with greater sperm production and higher ejaculate quality ([47,148]; reviewed in [80]). The variation we observed in scrotum width raises the possibility that testes size may change with dominance rank status, as has been reported in other species such as mandrills [81] and seasonally breeding birds ([149]; reviewed in [150]). However, these results invite further longitudinal studies to definitively determine the extent and nature of these changes. For instance, the observed variation could also be linked to differences in mating activity; specifically, alpha males, who mate more frequently, may develop larger scrotums as a necessary adaptation to produce more sperm.

Some species exhibit a trade-off between investment in traits that enhance pre-mating competition (weaponry and badges of status) and those beneficial for post-mating competition (testes size) [151]. For example, in polygynous gorillas (*Gorilla* spp.), males are twice as large as females but have disproportionately small testes relative to their body size. This indicates a strong emphasis on pre-mating competition and relatively low levels of post-mating competition [152]. In contrast, in species like capuchins, where female monopolization is more challenging, males must invest in traits that promote both pre- and post-mating competition [151]. The combination of visual badges of status (greater facial width and larger fWHR) and larger testes in male white-faced capuchins highlights the importance of both strategies for reproductive success. We suggest that the rank-based differences in facial morphology may serve as badges of status in this species, providing essential social and physical cues among males by reducing intra-group aggression and promoting cooperation during inter-group encounters, while larger testes give alpha males an additional edge in reproductive competition.

## 5. Conclusions and future directions

This study makes a significant contribution to the broader understanding of dominance-related phenotypes by providing quantitative evidence of how facial and testicular dimensions vary according to male dominance rank status in wild white-faced capuchins. Our findings lay a foundation for subsequent studies to examine how these phenotypes vary within individuals across different life-history stages, their role in social interactions and their ultimate impact on reproductive success.

Future studies should verify that the observed morphological differences function as signals of dominance by examining whether conspecifics perceive and respond to them. These data are needed to determine if these morphological traits are shaped by sexual selection and act as badges of status or are merely by-products of physiological factors like elevated androgen levels. Additionally, given the impermanence of male dominance status in many primate species, longitudinal studies are needed to track hormonal, behavioural and morphological changes before and after attaining high dominance rank to clarify the timing and directionality of these associations.

## Data Availability

Datasets and scripts are available from the Dryad repository [153]. Supplementary material is available online [154,155].
